# Adaptive Compressive Sensing and Data Recovery for Periodical Monitoring Wireless Sensor Networks

**DOI:** 10.3390/s18103369

**Published:** 2018-10-09

**Authors:** Jian Chen, Jie Jia, Yansha Deng, Xingwei Wang, Abdol-Hamid Aghvami

**Affiliations:** 1 Computer Science and Engineering, Northeastern University, Shenyang 110819, China; chenjian@mail.neu.edu.cn (J.C.); wangxingwei@mail.neu.edu.cn (X.W.); 2 Research Center of Safety Engineering Technology in Industrial Control of Liaoning Province, Neusoft Group Research, Shenyang 110179, China; 3 Key Laboratory of Computer Network and Information Integration (Southeast University), Ministry of Education, Nanjing 211189, China; 4 Department of Informatics, King’s College London, London WC2R 2LS, UK; yansha.deng@kcl.ac.uk (Y.D.); hamid.aghvami@kcl.ac.uk (A.-H.A.)

**Keywords:** adaptive compressed sensing, data recovery, step size determination, wireless sensor networks

## Abstract

The development of compressive sensing (CS) technology has inspired data gathering in wireless sensor networks to move from traditional raw data gathering towards compression based gathering using data correlations. While extensive efforts have been made to improve the data gathering efficiency, little has been done for data that is gathered and recovered data with unknown and dynamic sparsity. In this work, we present an adaptive compressive sensing data gathering scheme to capture the dynamic nature of signal sparsity. By only re-sampling a few measurements, the current sparsity as well as the new sampling rate can be accurately determined, thus guaranteeing recovery performance and saving energy. In order to recover a signal with unknown sparsity, we further propose an adaptive step size variation integrated with a sparsity adaptive matching pursuit algorithm to improve the recovery performance and convergence speed. Our simulation results show that the proposed algorithm can capture the variation in the sparsities of the original signal and obtain a much longer network lifetime than traditional raw data gathering algorithms.

## 1. Introduction

Wireless Sensor Networks (WSNs), which are capable of sensing, computing, and wireless communication, can be applied to a wide range of applications, such as scientific observation, emergence detection, climate detection, ecosystem surveillance, and physical hazard prevention [1]. In many of these applications, sensor nodes are powered by battery and deployed in an unattended hostile environment with high density. Once deployed, these nodes should send their sensing results to the sink node periodically. Due to the constraints of application environments, it is crucial to prolong the network lifetime of WSN. According to the energy consumption model presented in [2], the energy consumed in a sensor node is exponentially increased with the communication distance. As a result, sensor nodes usually follow the routine to the sink node via multihop transmission, thus saving energy. Besides that, multihop communication is essential for large-scale WSNs for cases where the transmission range of sensor nodes is much smaller than the size of the target area.

Since all the sensor nodes forward their data to only one sink node, the routing tree in WSNs usually exhibits a many-to-one structure, which is often called *convergecast* [3,4]. The main drawback of the *convergecast* structure is the energy hole problem, chiefly because the sensor nodes close to the sink node have to relay more packets and tend to run out of energy sooner. As a result, the entire network is subject to premature death because it is separated by the energy hole. In order to avoid uneven energy depletion and to extend the network’s lifetime, several advanced hardware and software solutions have been recently proposed. As for the design of hardware, wireless energy charging technologies are applied to harvest energy from ambient sources [5,6]. However, recharging is impossible or not worth it in many applications [7]. As for the design of software, data gathering solutions have been proposed to reduce the traffic load. Since the physical phenomena collected by sensor nodes often possesses strong temporal and spatial correlations, it is inefficient to deliver raw data to the sink node. Many solutions have incorporated data correlations into data gathering, such as distributed source coding [8] and the distributed compression algorithm [9]. However, conventional data compression techniques, which are usually associated with the design of routing, require heavy computation and communication loads on sensor nodes [10]. Source coding techniques are also incompatible with WSNs, since they work with the assumption that the statistical structure of the underlaying data distribution should be known prior [11].

The emerging technology of compressive sensing (CS) opens up a new perspective for data gathering in WSNs. The basic idea of CS is that if the signal is sparse or compressible at a certain level , it can be reconstructed from a small number of linear measurements lower than the Shannon–Nyquist limit [12]. The application of CS for data gathering in multi-hop WSNs has drawn much attention recently. [13] investigated how to apply CS theory for data gathering. They also designed a simple but efficient measurement matrix that satisfies the restricted isometry property (RIP). CS based techniques can substantially reduce the amount of data transmission and balance the traffic load throughout the entire network with a very low level of complexity. In order to further reduce the energy consumption and improve the energy efficiency, a novel measurement matrix generation incorporating a transmission design was proposed in [14]. In [15], the CS principle was used as a compression and forwarding scheme to minimize the transmission data. In Ref. [16], CS based data aggregation combined with routing was designed to reduce the entire energy consumption. In Ref. [17], a CS method of cluster-based WSNs was designed, and centralized and distributed clustering methods were proposed to reduce the number of transmissions. Considering the spatial correlations between sensor nodes, the compressive sleeping strategy was designed in Refs. [18,19] for high density WSNs, where only a subset of sensor nodes were active and all the other sensor nodes were turned off to save the energy and extend the network’s lifetime. By utilizing the spatial correlations of the data in densely deployed WSNs, a distributed compressive sensing scheme for data gathering was proposed in Ref. [20], where the belief propagation algorithm was employed for signal recovery. In Ref. [21], the spatial interpolation method was proposed to transform a predetermined sparsifying measurement matrix for WSNs with only local communications, avoiding the complete position knowledge of the entire network being obtained. In Ref. [22], the CS based signal and data acquisition for WSNs as well as the Internet of Things (IoT) was proposed, and a cluster-sparse reconstruction algorithm was proposed for in-network compression to achieve accurate signal recovery and energy efficiency. By exploiting a similar sparsity structure of acoustic signals from nodes in the same array, a collaborative reconstruction method was proposed for data gathering in wireless sensor array networks [23].

Even though various applications of CS for data gathering have been extensively studied, they all assume that the sparsity of the signal is static or changing sufficiently slowly over time. However, such an assumption is too restrictive for many monitoring applications, where the sparsity of the signal changes rapidly with time or conditions. For example, for an anti-fire forest monitoring WSN, due to the fact that the number of measurements is linearly related to the underlying signal sparsity level, the signal sparsity with and without fire is apparently different. When the sparsity of the signal is changed, ignoring the rapid change of signal sparsity will result in performance degradation in signal recovery. Therefore, it becomes important to design an adaptive compressive sensing in accordance with the variation of the signal sparsity.

After gathering the data with compressive sensing, another challenge is how to design a signal recovery algorithm with fast reconstruction and reliable accuracy. Recently, the signal recovery algorithm applied in WSNs was classified into two categories: (1) the basis pursuit (BP) which was proposed to find the $l_{1}$ minimization using linear programming, and (2) the iterative greedy pursuit, for example, the orthogonal matching pursuit (OMP), the stagewise OMP (StOMP) [24] and the compressive sampling matching pursuit (CoSaMP) [25]. Among them, BP requires the least number of measurements; however, its high computational complexity prevents it from being used for large scale applications. OMP and StOMP adopt a bottom-up approach in signal recovery, and their complexity levels are much lower than that of BP. However, they require more measurements and have a lack of recovery guarantee. CoSaMP adopts a top-down approach and can offer an acceptable recovery performance, similar to that of the BP method, but with a much lower recovery complexity. However, all these algorithms discussed above assume that the sparsity of the signal is known prior. These algorithms cannot be directly applied to the construction of a signal with unknown sparsity. For blind signal recovery with unknown sparsity, the most commonly applied algorithm is the sparsity adaptive matching pursuit (SAMP) [26], where the sparsity of signal and the true support of the signal is estimated stage by stage with the “divide and conquer” method. SAMP can also be viewed as a generalization of existing algorithms, such as OMP or CoSaMP. However, the design of an appropriate step variation to guarantee fast convergence and accuracy is still an open problem.

Different from aforementioned works, the aim of this work was to design an adaptive compressive sensing framework for periodical monitoring of WSNs. As we know, the most related work to this research is the intelligent compressive sensing scheme proposed in Refs. [27,28]. The main difference between our work and their works is the sparsity determination. In Ref. [27], sparsity was obtained with local correlations, and the signal was recovered by successive reconstruction applied at the sink node. In Ref. [28], the compressive sampling was applied in a single-hop IoT system, and a learning phase was designed at the central smart object to select the sparsity level. However, in our work, compressive sampling is applied in multi-hop wireless sensor networks, and the variation in the sparsity is determined by very few re-sampling iterations . Additionally, we present an improved SAMP to recover signal with unknown sparsity. The main contributions of this paper are summarized as follows:

- We propose an adaptive compressive sensing framework for periodical monitoring of WSNs, where a reconstruction error estimation module is designed to check whether the current sampling rate is still sufficient for signal reconstruction, and a sparsity determination module is designed to estimate the sparsity and calculate the required sampling rate at the next monitoring period.
- We propose an efficient sparsity variation determination algorithm, which can determine the current sparsity as well as the new sampling rate by only re-sampling a few measurements to save the energy cost and guarantee the recovery performance.
- We propose an improved SAMP algorithm to recover the signal with unknown sparsity, where both the linear and non-linear step size variation are designed to guarantee fast convergence and reliable accuracy.
- We evaluate the proposed algorithms with extensive simulations and study the impacts of multiple environmental factors, including the number of sensors and the different sampling rates. The simulation results show that our proposed algorithm could achieve substantial improvements compared with existing algorithms in terms of sparsity matching and signal recovery.

The remainder of this paper is organized as follows. Section II presents the mathematical details for the CS and data gathering. We propose our adaptive signal sampling method with unknown sparsity in Section III. The signal recovery algorithm is presented in Section IV. Section V presents the numerical results, and Section VI concludes the paper.

## 2. Data Gathering Based on Compressive Sensing

We consider a monitoring WSN consisting of *N* sensor nodes and a sink node, where the set of sensor nodes is denoted as N={1,...,N}, and the only sink node is denoted as *S*. We denote the monitoring period as *P*. Sensor nodes are distributed in the target area to sense the physical conditions and report the sensory reading to the sink node in each period (p∈P).

It is assumed that the sensed data for sensor node *i* (i∈N) in time period *p* is $x_{i,p}$, and the signal of all the sensor nodes in time period *p* can be represented by an *N*-dimensional vector, $x_{p}={\left(x_{1,p},\cdots ,x_{N,p}\right)}^{T}$. It is said that $x_{p}$ is a *k*-sparse signal in domain $\Psi_{p}$ if $x_{p}$ can be represented by a *k*-sparse vector, $d_{p}$, in domain $\Psi_{p}$, and is given by

(1) $$x_{p}=\sum_{i=1}^{N}d_{i,p}\psi_{i,p}=\Psi_{p}d_{p}$$

where $\Psi_{p}=\left[\psi_{1,p};\cdots ;\psi_{N,p}\right]$ ($\psi_{i,p}\in R^{N}$) is the orthonormal basis, and $d_{p}$ represents the transform coefficients with only *k* non-zero values.

The compressive sensing theory states that an *N*-dimensional signal with *k*-sparsity can be represented by *M*(M<N) linearly projected measurements. Let Φ (M×N) be the measurement matrix, $x_{p}$ can be given as

(2) $$y_{p}=\Phi x_{p}=\Phi \Psi_{p}d_{p}.$$

According to Ref. [29], the exact recovery of $x_{p}$ can be achieved through solving the following combinatorial optimization problem:

(3) $$\min_{x\in R^{N}}\left|\right|X_{p}{\left|\right|}_{l_{0}}\;\;\;s.t.\;y_{p}=\Phi x_{p}.$$

This is an NP-hard problem, and it is hard to obtain the optimal solution. However, it is equivalent to the following $l_{1}$ optimization problem if the incoherence property between Φ and Ψ or the restricted isometry property (RIP) [30] of matrix ΦΨ satisfies

(4) $$\min_{d\in R^{N}}\left|\right|d_{p}{\left|\right|}_{l_{1}}\;\;\;s.t.\;y_{p}=\Phi \Psi d_{p},x_{p}=\Psi_{p}d_{p}.$$

The above 1-minimization problem is more tractable and can be solved with linear programming (LP) techniques [31]. We can further obtain the recovered signal, $\hat{x_{p}}$, with the known orthonormal basis, $\Psi_{p}$.

**Figure 1:**
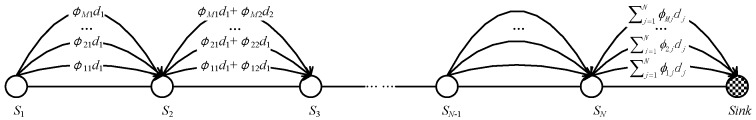
Data gathering based on compressive sensing.

With the above discussion, it is clear that applying CS to WSNs relies greatly on two important issues: First, how to choose or find an efficient orthonormal basis to represent the original signal. Generally, sensor readings are spatially smooth and sparse in the frequency domain; thus, we can use the wavelet transform or the discrete cosine transform can be applied to add sparsity to the original signal [14]. However, for signals with abnormal reading or transmission errors, it is hard to add sparsity in the frequency domain. To cope with this, sparse representation based on overcomplete dictionaries has been proposed [32]. With an overcomplete dictionary that contains prototype signal-atoms, signals are described by sparse linear combinations of these atoms. Various dictionary learning methods have been proposed in the literature. For example, the original (synthesis) K-SVD is one such method which allows the construction of an overcomplete dictionary that is suitable for sparse synthesis by learning the dictionary from the data itself [32]. After find a sparsity method, another problem in designing compressive sensing is how to design a measurement matrix (Φ) such that a good RIP is attained. It has been shown that a random matrix with Gaussian variables complying to $N(1,\frac{1}{M})$ has a good RIP [33]. Algorithm 1 illustrates the process of compressed sampling with sampling rate *M* in detail. In this algorithm, each time a sensor (*i*) in period *p* has a value $x_{i,p}$ to transmit to the sink node, it first calculates a new value ($y_{i,p}^{m}$) by multiplying value $x_{i,p}$ with a Gaussian variable, $\phi_{i,p}^{m}$ (1≤m≤M). Then, this new value ($y_{i,p}^{m}=\phi_{i,p}^{m}x_{i,p}$) is aggregated and transmitted along the path to the parent node (*j*). In this way, the transmitted data of sensor node *j* is the summation of $y_{j,p}^{m}$ and the value from all its children ($\sum_{i\in c_{j}}y_{i,p}^{m}$), where $c_{j}$ is the set of children of sensor node *i*. Finally, the sink node receives the value as $\sum_{n}\phi_{i,p}^{m}x_{i,p}$. This process is repeated until *M* linearly projected measurements are obtained, as shown in Figure 1. Similarly to Refs. [14,17,34], we assume the measurement coefficient $\phi_{i,p}^{m}$ is generated using a pseudorandom number generator seeded with the identifier of node *i*, which means that the measurement matrix can be easily constructed locally at the node itself.

**Algorithm 1:** Compressive sampling with sampling rate *M*

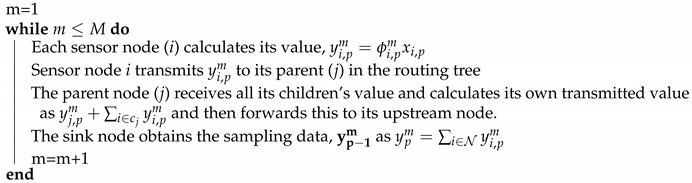



According to the CS theory in Ref. [33], when the number of measurements (*M*) satisfies M∝ck with a constant (*c*), the original signal with *k*-sparsity can be recovered with high probability. Due to the fact that the sparsity of a signal usually satisfies k<<N, it is clear that the amount of data transmission in CS-based data gathering is much smaller than that in these traditional methods; thus, reducing the communication costs and achieving energy efficiency during data gathering.

## 3. Adaptive Sampling for Signals with Dynamic Sparsity

**Figure 2:**
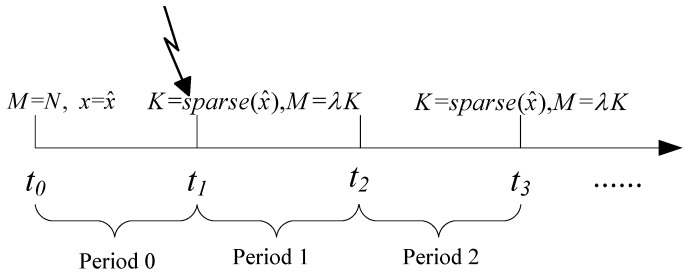
Periodical monitoring with data re-sampling at the start of each period.

From the description of the CS based data gathering, it can be known that for a signal with *k*-sparsity, the optimal sampling rate should be at least ck to guarantee the recovery accuracy and to save communication energy. However, for a signal with unknown sparsity, it is very challenging to select the optimal sampling rate to match the sparsity. In this section, we therefore propose an adaptive sampling mechanism for signals with variable sparsity. In our approach, we assume that the sparsity of the signal in period *p*, denoted as $k_{p}$, remains fixed during the entire period, as shown in Figure 2. However, at the starting time of the next period, its sparsity is re-examined, and the new sampling rate is determined corresponding to its new sparsity.

It should be noted that at the starting time of period p+1, it is hard to decide whether the previous sampling ($\alpha_{p}$) still matches the sparsity of the signal at period p+1. A straightforward solution is to conduct raw data gathering with all active sensor nodes. This approach, however, results in the decrease of the network’s lifetime and is inefficient for networks with larger numbers of sensor nodes. Therefore, in order to determine the minimum sampling rate required to recover a signal with unknown sparsity, a compressive sampling method based on sequential observations is proposed.

${\hat{x}}^{M}$ is denoted as the recovered signal with sampling rate *M*. When noiseless measurements are taken using the random Gaussian ensemble, we have the following lemma [35].

**Lemma** **1.** *For a Gaussian measurement ensemble, if ${\hat{x}}^{M+\alpha }={\hat{x}}^{M}$, then we can conclude that ${\hat{x}}^{M}=\hat{x}$ with a probability of 1.*

According to Lemma 1, the recovered signals with *M* samples and M+α samples are first compared. If the samples match, we declare that these signals are correctly recovered. In a practical setting, most of the coefficients after orthogonal transformations are relatively small, rather than being exactly zero, which means only approximate sparseness is obtained. Thus, the recovery error between the original signal and the recovery with *M* sequential samples is given by [35]

(5) $$\left|\right|\hat{x}-{\hat{x}}^{M}{\left|\right|}_{2}<\frac{\left|\right|{\hat{x}}^{M}-{\hat{x}}^{M+\alpha }{\left|\right|}_{2}}{\sin\theta }$$

where θ is the angle between vectors $\hat{x}$ and ${\hat{x}}^{M}$, and has the following approximation

(6) $$E\left(\frac{1}{\sin\theta }\right)\geq \left|\right|{\hat{x}}^{M}-{\hat{x}}^{M+\alpha }{\left|\right|}_{2}\sqrt[{}]{\frac{N-M}{\alpha }}.$$

The recovery error can be further approximated as

(7) $$\left|\right|\hat{x}-{\hat{x}}^{M}{\left|\right|}_{2}\approx \left|\right|{\hat{x}}^{M}-{\hat{x}}^{M+\alpha }{\left|\right|}_{2}\sqrt[{}]{\frac{N-M}{\alpha }}.$$

In our paper, the recovery error between ${\hat{x}}^{M}$ and ${\hat{x}}^{M+\alpha }$ is applied to determine the variation of the sparsity, and an adaptive sampling approach is proposed for the periodical monitoring of sensor networks.

**Figure 3:**
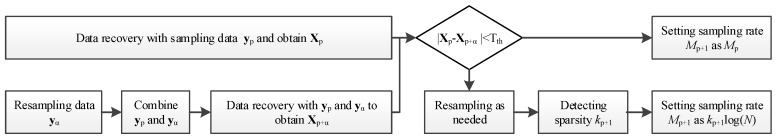
The process of adaptive sampling.

In Algorithm 2, the process of the adaptive sampling is illustrated in detail. In this algorithm, $M_{p}$ is denoted as the sampling rate at period *p*. Initially, raw data gathering is applied, to obtain accurate sparsity of the entire signal. After that, at the starting time of period *p*, the sampled data obtained at period $p_{1}$, denoted as $y_{p-1}^{M_{p}}$, is recovered as ${\hat{x}}^{M}$. Meanwhile, a data re-sampling operation is executed to get re-sampled data ($y_{p}^{\mathrm{ff}}$) with a fixed number of sampling points as α. After re-sampling, $y_{p}^{\mathrm{ff}}$ and $y_{p-1}^{M_{p}}$ are further combined as $y^{M+\mathrm{ff}}$ and recovered as $y^{M+\mathrm{ff}}$ at the sink node. Therefore, the performances of $y_{p+\mathrm{ff}}$ and $y_{\mathrm{ff}}$ are compared to decide whether the gap between them is larger than the predefined threshold ($T_{th}$). If it is detected that the recovery gap is smaller than $T_{th}$, it is concluded that the sparsity is the same as that in period $p_{1}$, and the sampling rate in period *p* is set as $M_{p}=M_{p-1}$. If it is detected that the recovery gap is larger than $T_{th}$, the novel sparsity is calculated. Due to fact that re-sampling with a fixed sampling rate (α) may be unable to recover the original signal, extra sampling with sampling rate $\alpha_{s}$ is executed, where $\alpha_{s}=\beta ||{\hat{x}}^{M}-{\hat{x}}^{M+\alpha }{\left|\right|}_{2}-\alpha$, and β is a scale coefficient related to the recovery gap. From the expression of $\alpha_{s}$, it is known that the larger the gap is, the more re-sampling iterations needed to obtain its new sparsity level. After that, the sampling rate for period *p* is determined and broadcast to all the sensor nodes. Similar to Refs. [14,17], it is assumed that the signal recovery and sparsity estimation are conducted at the sink node, and only the determined sparsity is sent back to each sensor node, thus reducing the energy consumption of each sensor node. It should be noted that our algorithm adopts the same data gathering procedure (shown in Algorithm 1) as that of traditional compressive based methods. The only difference lies in the degree to which the sampling rate corresponds with the signal variation, which means that the data transmission delay is almost the same. Compared with raw data gathering, the time consumed in compressive data transmission is much longer, which is mainly because compressive based data gathering needs to occur on the same date *M* times. However, it should be noted that compressive based data gathering obtains balanced energy consumption among all sensor nodes, and has a much longer network lifetime than raw data gathering. For clarity, Figure 3 illustrates the detailed process of how to decide the sparsity at period *p*.

**Algorithm 2:** Adaptive compressive sampling

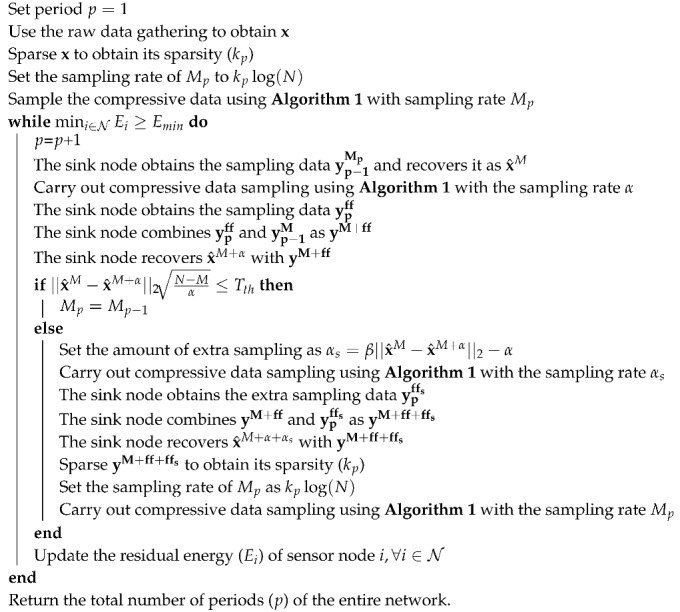



## 4. Signal Recovery with Unknown Sparsity

In sequential-based data gathering, signal recovery requires the use of non-linear algorithms to find the sparsest signal from the measurements. One challenging question in CS research is the design of a fast reconstruction algorithm with reliable accuracy and (nearly) optimal theoretical performance. The existing signal recovery algorithms always require the signal sparsity first. For signals with unknown sparsity, the sparsity adaptive matching pursuit (SAMP) has already been widely used to recover many blind signals with unknown sparsity.

**Figure 4:**
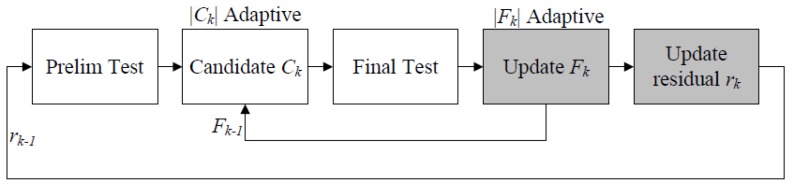
A conceptual diagram of SAMP.

In contrast to other state-of-the-art greedy algorithms, SAMP takes advantage of both the “bottom-up” approach and the “top-down” approach, where the “bottom-up” method is applied to estimate the sparsity of the signal, step-by-step, and the “top-down” method is applied to identify the true support of the signal by backtracking strategy. Figure 4 shows the conceptual diagram of SAMP, and it can be observed that the sizes of the candidate set ($|C_{k}|$) and finalist ($|F_{k}|$) are adaptive. However, the recovery accuracy and speed of SAMP highly relies on the step size (*s*). In order to avoid overestimation, the safest choice is to set *s* as 1 for an unknown *k*, but many more iterations are needed for convergence. How to provide a recovery accuracy with fast convergence speed has become a major challenge.

In this paper, we propose an adaptive step decision that corresponds with the number of iterations. The step size at iteration *t* is denoted as $s_{t}$, which is given by

(8) $$s_{t}=s_{t}+\omega_{t}\Delta_{t}$$

where $\omega_{t}$ is the weight factor employed to regulate the trade-off between the speed and accuracy, and $\Delta_{t}$ is the fixed step difference between any two adjacent iterations.

In our paper, two step variation approaches are proposed: the linear decrement weight factor and the non-linear decrement weight factor. For the linear weight factor decrement approach, the weight factor is given by

(9) $$\omega_{t}=\omega_{max}-\frac{\omega_{max}-\omega_{min}}{T_{max}}t$$

where $\omega_{max}$ and $\omega_{min}$ are the maximum and minimum weight factors, and $T_{max}$ is the maximum number of iterations. For the non-linear weight factor decrement approach, the weight factor is given by

(10) $$\omega_{t}=\omega_{max}-{\left(\frac{t-1}{T_{max}-1}\right)}^{\lambda }\left(\omega_{max}-\omega_{min}\right)$$

where the parameter λ is applied to control the convergence speed, and $\omega_{t}$ decreases with an increase in λ. In constrast to the linear weight factor decrement, the decrease in the step size is much smaller than that with the linear method, reducing the possibility of getting the local optimal solution.

Algorithm 3 presents the detailed pseudo code of the proposed recovery algorithm with an adaptive step. Here, $\Phi^{*}$ represents the transpose of matrix Φ, $\Phi^{†}$ represents the Moore–Penrose inverse of matrix Φ, *s* represents the step size of the finalist, and the function Max(F,s) returns *s* elements corresponding to the largest absolute value of vector F. Additionally, for a set Λ={1,2,⋯,N}, $\Phi_{\Lambda }$ is the sub-matrix of Φ with indices (i∈Λ). At the *s*-th iteration, $S_{t}$, $C_{t}$, $F_{t}$, and $r_{t}$ denote the shortlist, the candidate list, the finalist, and the observation residual. In this paper, the maximum iteration time ($T_{max}$) is set as the stopping rule, and the recovering threshold (ϵ) is applied as the halting condition.

**Algorithm 3:** Adaptive compressive sampling

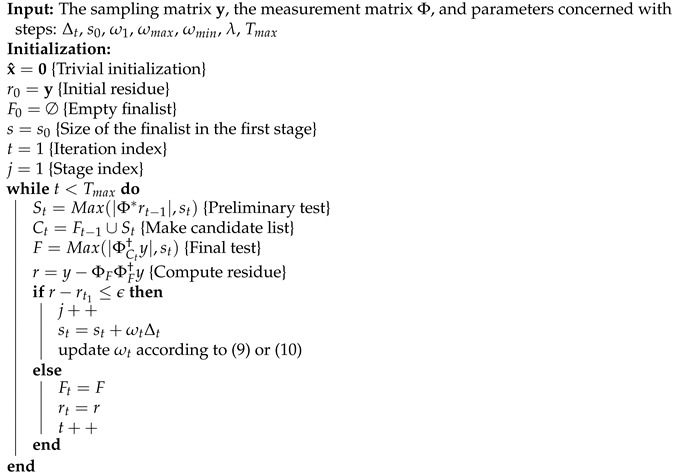



## 5. Numerical Results

In this section, we provide numerical results to illustrate the performance of our proposed algorithm. We consider a WSN with a fixed sink node and no more than 400 sensor nodes randomly deployed in a square area of size 500 × 500. It is assumed that the sink node is located in the center of the area. The initial energy of each sensor node was set to 10,800 J. The corresponding simulations were implemented in Matlab R2009a using a laptop with an Intel (i5-4300) CPU. All the results were obtained by averaging over 100 simulations.

### 5.1. Sparsity Analysis

**Figure 5:**
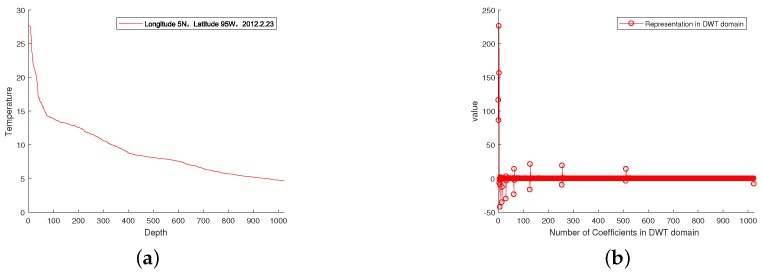
(**a**) Raw signal from monitoring the sea temperature, (**b**) sparse analysis of the raw signal in the Discrete Wavelet Transform (DWT) domain.

**Figure 6:**
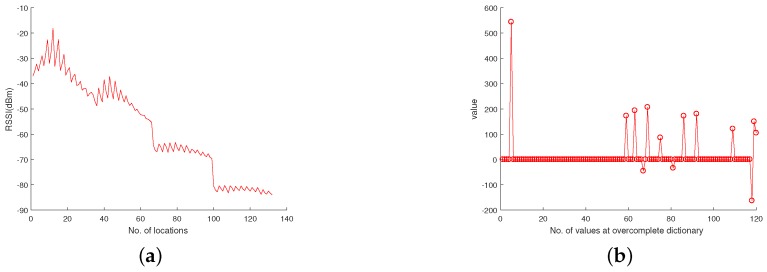
(**a**) Raw RSSI signal of a smartphone from a real environment, (**b**) sparse analysis of the raw signal based on the K-SVD algorithm.

First, we took the raw signals from the real ocean temperature monitored by the National Oceanic and Atmospheric Administration (NOAA) for the sparsity analysis. For instance, Figure 5a shows the sea temperature monitoring data collected at half past 6, February 23, 2012 in the location of 5 N, 95 W. This data contains 1040 temperature measurements at different depths. From Figure 5a, it can be observed that the signal in the time domain is not sparse. We therefore used the Discrete Wavelet Transform (DWT) to find its sparsity. As shown in Figure 5b, it can be observed that the raw data has good sparsity in the DWT domain—it only has 76 non-zero values. We thus conclude that it is a 76-sparse signal in DWT domain. We further took a raw RSSI measurement of an access point (AP) from a smartphone detected in a real environment [36]. Due to the channel-path fading and interference from nearby equipment, it was hard to find an orthonormal basis from the DWT domain, as shown in Figure 6a. We thus applied the K-SVD algorithm based on an overcomplete dictionary to sparse it. Figure 6b shows that the raw data has good sparsity with the K-SVD algorithm. It has 13 non-zero zones, which also showcase that the RSSI value is concentrated on 13 parts.

**Figure 7:**
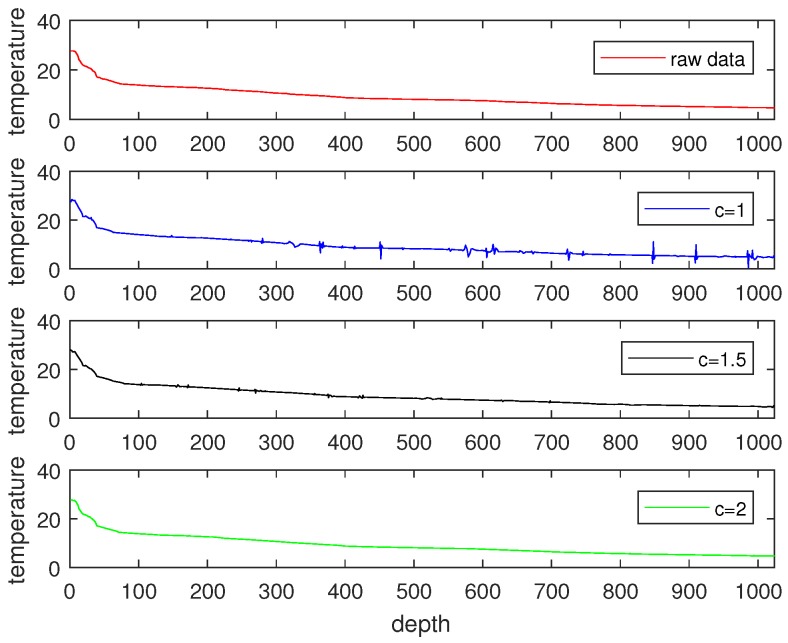
Signal recovery with a sparsity of 76 with different sampling rates.

**Figure 8:**
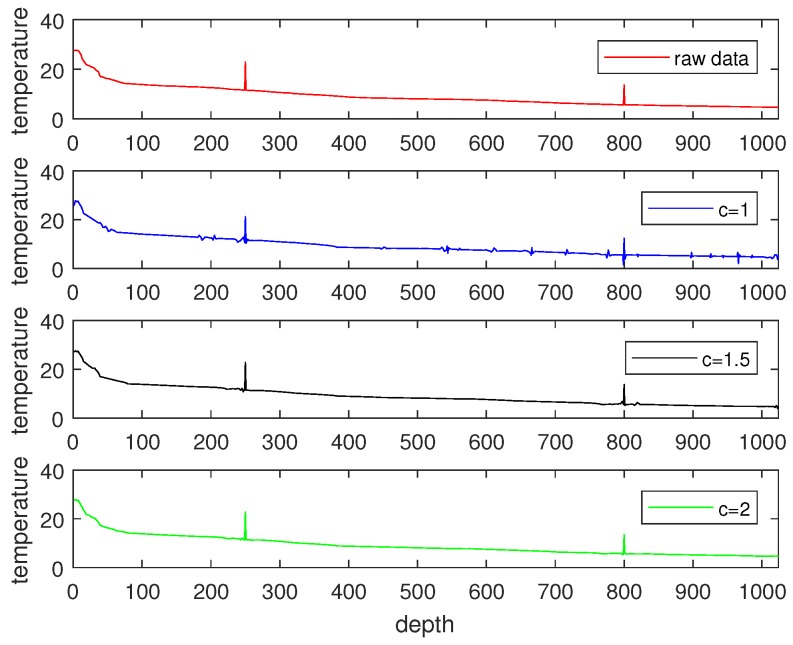
Signal recovery with a sparsity of 122 with different sampling rates.

**Figure 9:**
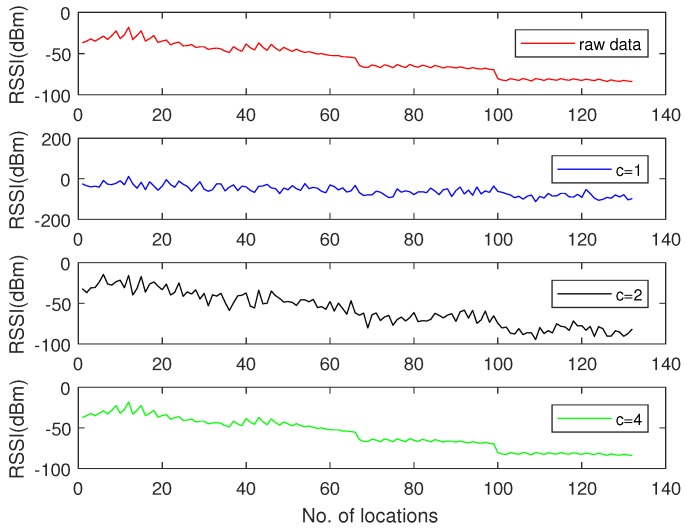
Signal recovery of RSSI data with different sampling rates.

Considering that signal sampling rate greatly relies on signal sparsity, and the number of sampling times *M* should satisfy M∝ck, we compared the signal recovery performance with different *c* values. The measurement matrix Φ was constructed by creating an M×N matrix with i.i.d. draws of a Gaussian distribution (N(0,1)). The recovered signals with different sampling rates are shown in Figure 7, Figure 8 and Figure 9, where Figure 7 illustrates the signal recovery with a sparsity of k=76 in the DWT domain, Figure 8 illustrates the signal recovery with a sparsity of k=122 in the DWT domain, and Figure 9 illustrates the signal recovery of the RSSI data with an unknown sparsity in the DWT domain, but it can be sparsed with an overcomplete dictionary with a sparsity of k=13. From these figures, it can be observed that as the signal recovery performance increases, the sampling rate *c* increases . This can be explained by the fact that a larger sampling rate *c* results in a larger measurement matrix (Φ), and thus, more energy is consumed in data gathering. Besides that, it was observed that for signals sparsed at the frequency domain, the recovered signal is almost the same as the raw data when the sampling rate is larger than 2k. However, for signals with an unknown sparsity at the frequency domain, the sampling rate should be set larger than 4k. Therefore, in this paper, when a signal could be sparsed at the frequency domain, and its sparsity was *k*, we set the sampling rate as 2k, whereas for signals that could not be sparsed at the frequency domain and for which the sparsity (*k*) was detected at the overcomplete dictionary, we set the sampling rate as 4k. With this scheme, for the signals shown in Figure 7 and Figure 8, the sampling rates were set as 2×76 and 2×122, which means the size of the measurement matrices (Φ) were 152×1024 and 244×1024. For the signals shown in Figure 9, the sampling rate was set as 4×13, and the size of its corresponding measurement matrix (Φ) was 52×132, respectively.

### 5.2. Adaptive Sampling

**Figure 10:**
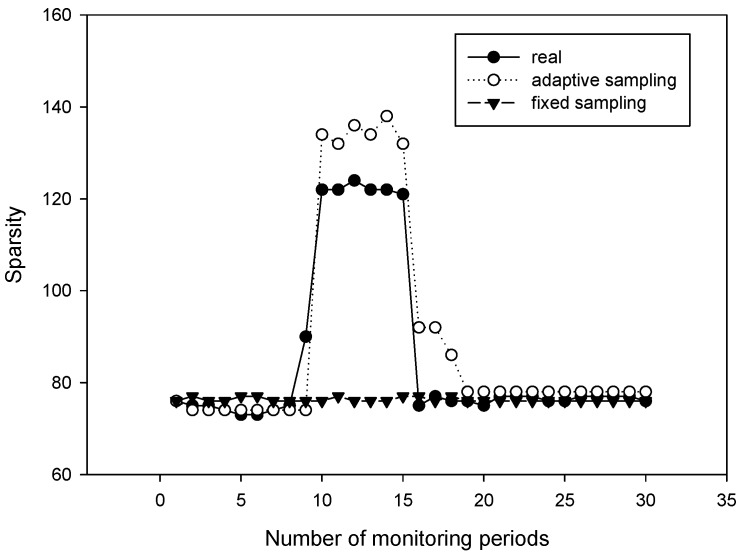
Sparsity estimation with different period numbers.

**Figure 11:**
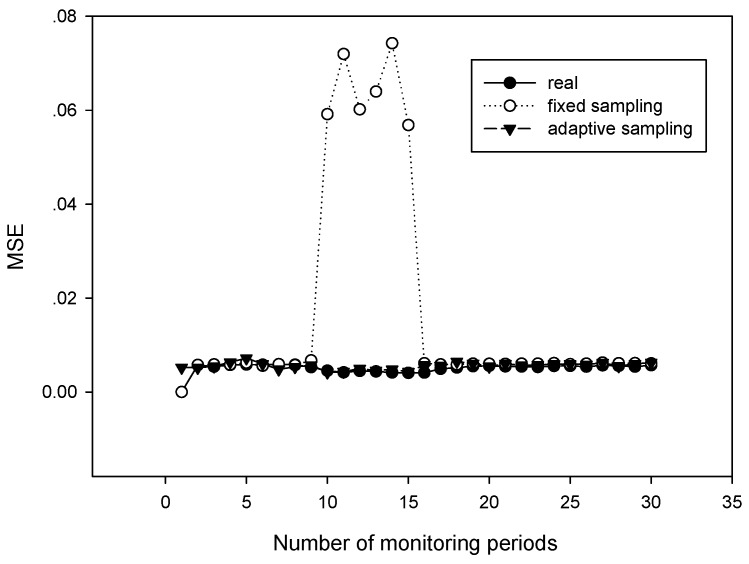
Signal recovery comparison within different period numbers.

In this simulation, we tested the performance of our algorithm for signals with dynamic sparsities. We considered a 200-node wireless sensor network, and applied Dijkstra as the routing algorithm. We compared our algorithm with the fixed sampling algorithm proposed in Ref. [13]. Figure 10 plots the sparsity estimation in the first 30 monitoring periods. Figure 11 plots the recovery performance in the first 30 monitoring periods. From Figure 10, it can be observed that during the time between the 10th and 15th periods, the sparsity of the signal changed. However, the fixed sampling method still uses a sampling rate settled at the initial state and cannot guarantee the required recovery performance. Compared with the fixed sampling algorithm, our algorithm always captured the variation in the sparsity; the estimated sparsity was almost the same as the real sparsity. Our algorithm obtained a much lower recovery error rate despite the changes in the environment, which shows that it is important to apply an adaptive sampling rate for the periodical monitoring of sensor networks.

**Figure 12:**
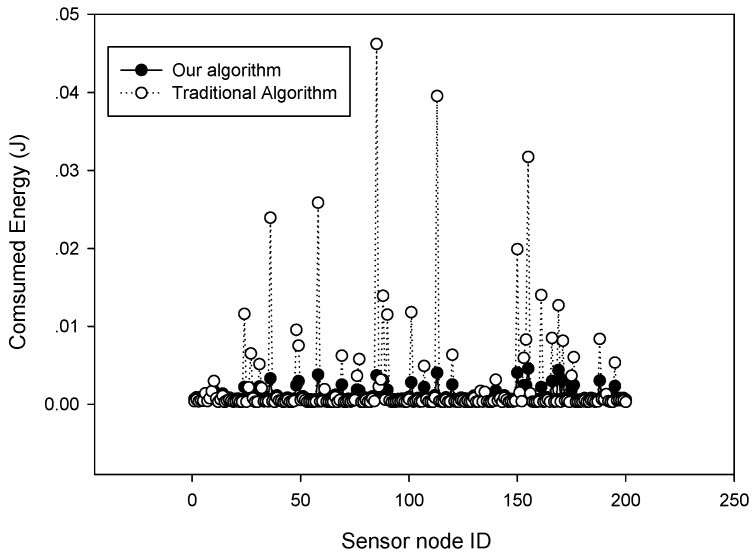
Energy consumption in one working period.

**Figure 13:**
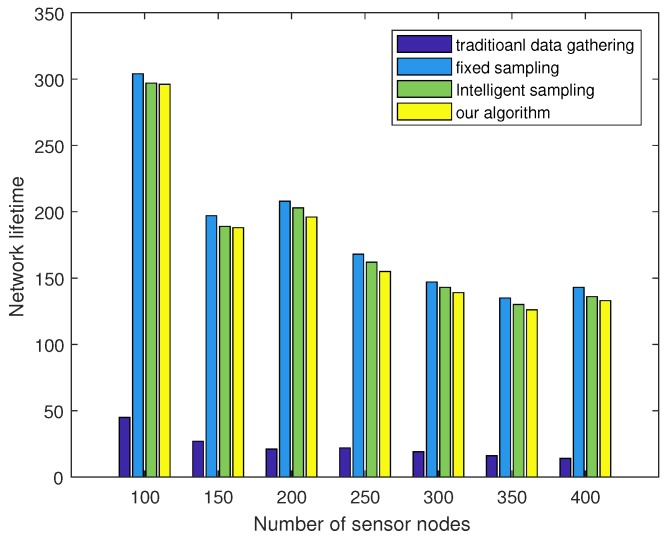
Network lifetime comparison with different numbers of sensors.

Considering that α extra data sampling is needed to estimate the sparsity in each period, we further tested the energy consumption and network lifetime performances and compared them with other algorithms. Here, the intelligent sampling represents the algorithm proposed in Ref. [27]. Figure 12 plots the energy consumption of each node in the 200-node sensor network in one working period. It can be observed that our algorithm obtained more balanced energy consumption compared with raw data gathering algorithm. Figure 13 plots the network lifetime comparison versus the number of sensor nodes. It can be observed that our algorithm achieved nearly the same network lifetime as that of fixed sampling or intelligent sampling. This can be explained by the fact that although adaptive sensing requires extra data gathering at the start time of each monitoring period, its sampling rate may be lower than the other two algorithms during the following sampling period. It can also be observed that our algorithm obtained a much longer network lifetime than that of raw data gathering, which shows that compressive sensing is an efficient data gathering method in wireless sensor networks.

### 5.3. Signal Recovery

**Figure 14:**
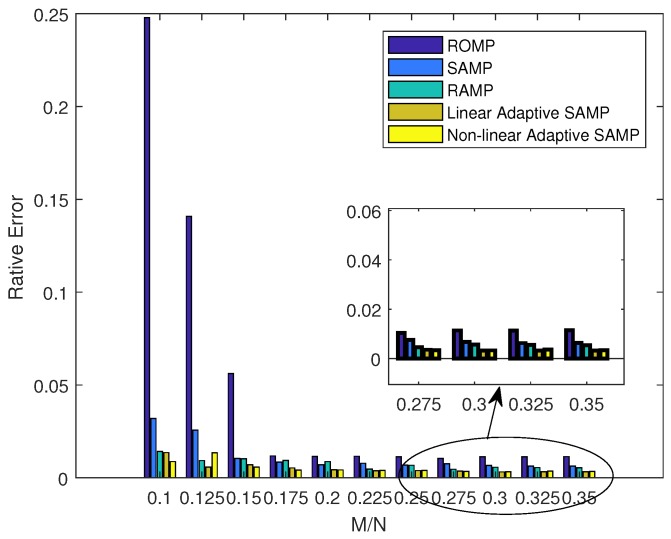
Comparison of the relative error with different sampling rates.

**Figure 15:**
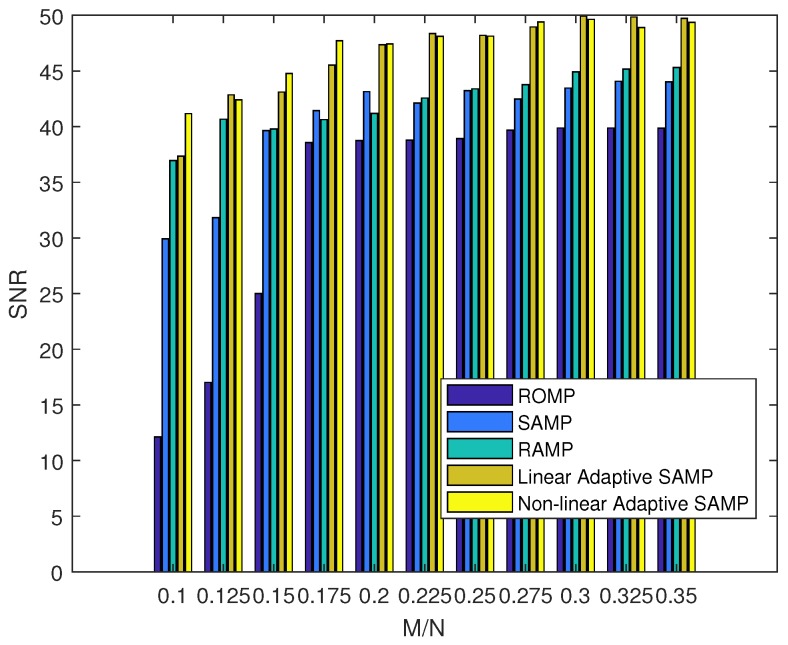
Comparison of the signal-to-noise ratio (SNR) with different sampling rates.

This simulation presents the signal recovery performance with unknown sparsities in a 200-node sensor network. Our algorithm was compared with the regularized orthogonal matching pursuit (ROMP), SAMP, and regularized adaptive matching pursuit (RAMP) [37]. We evaluated the reconstruction performance by using the averaged relative error (Re) and signal-to-noise ratio (SNR), where the relative error was defined as the average of Re=||x||2||x^−x||2 over 100 trials, and SNR was defined as $\mathrm{SNR}=10\log_{10}Re$. Figure 14 and Figure 15 plot the signal recovery performance with different algorithms. It was observed that the signal recovery performance can be substantially improved with an increase in sampling rate. It was also observed that our algorithm obtained a similar performance with linear or non-linear factors. Table 1 further shows a comparison of the convergence times. From this table, it can be observed that our algorithm took no more than 0.5 s to converge for the optimal solutions, while the traditional SAMP with step = 1 (denoted as SAMP-1 in Table 1) needed nearly 0.5 s and the SAMP with step = 3 (denoted as SAMP-3 in Table 1) needed about 0.1 s. This shows that our algorithm not only improves the recovery performance, but also converges much faster than the traditional SAMP method.

**Figure 16:**
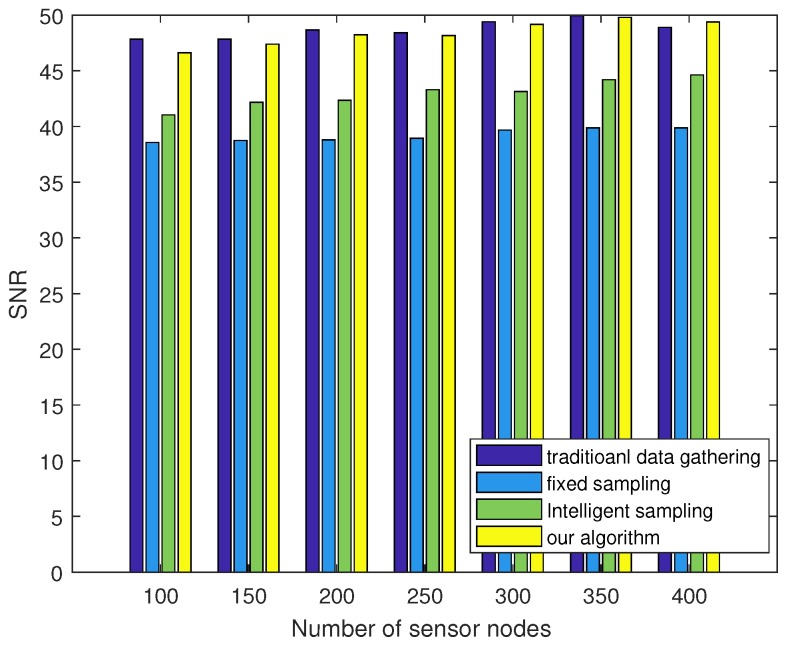
Comparison of SNR with different numbers of sensors.

We further tested the recovery performance with different sensor deployment densities and compared it with different sampling algorithms. Figure 16 plots the SNR versus the number of sensor nodes. Combined with the results shown in Figure 13 and Figure 16, it can be concluded that although our algorithm obtained a lower network lifetime compared with other compressive sampling methods, it obtained the best recovery performance. In addition, the SNR value was almost the same as that of raw data gathering. However, our algorithm was much more energy efficient than that of raw data gathering.

## 6. Conclusions

In this paper, we presented an adaptive sampling data gathering scheme for the periodical monitoring of wireless sensor networks. We developed a sequential observation based scheme to observe the variation in sparsity with fewer re-sampling measurements. We designed an adaptive construction step determination to improve the performance of SAMP in which both linear and non-linear step variation were designed to guarantee fast convergence and accuracy. Our simulation results demonstrate that our algorithm can efficiently capture the sparsity variation, and obtain greater recovery performance compared with existing compressive sensing methods with fixed sampling or intelligent sampling. It also obtains a much longer network lifetime than the traditional data gathering algorithm.

## Figures and Tables

**Table 1:** Convergence time comparison.

Algorithm	ROMP	SAMP-1	SAMP-3	RAMP	LA-SAMP	nLA-SAMP
Time(s)	0.344	1.622	0.542	0.453	0.437	0.438

## References

[1] Yao Y., Cao Q., Vasilakos A.V. (2015). EDAL: An energy-efficient, delay-aware, and lifetime-balancing data collection protocol for heterogeneous wireless sensor networks. IEEE ACM Trans. Netw..

[2] Ren J., Zhang Y., Zhang K., Liu A., Chen J., Shen X.S. (2016). Lifetime and Energy Hole Evolution Analysis in Data-Gathering Wireless Sensor Networks. IEEE Trans. Ind. Inform..

[3] Azam I., Javaid N., Ahmad A., Abdul W., Almogren A., Alamri A. (2017). Balanced Load Distribution With Energy Hole Avoidance in Underwater WSNs. IEEE Access.

[4] Jia J., Wu X., Chen J., Wang X. (2012). Exploiting sensor redistribution for eliminating the energy hole problem in mobile sensor networks. Eur. J. Wireless Commun. Netw..

[5] Deng Y., Wang L., Elkashlan M., Renzo M.D., Yuan J. (2016). Modeling and Analysis of Wireless Power Transfer in Heterogeneous Cellular Networks. IEEE Trans. Commun..

[6] Jia J., Chen J., Deng Y., Wang X., Aghvami A.H. (2017). Joint Power Charging and Routing in Wireless Rechargeable Sensor Networks. Sensors.

[7] Liu X.Y., Zhu Y., Kong L., Liu C., Gu Y., Vasilakos A.V., Wu M. (2015). CDC: Compressive Data Collection for Wireless Sensor Networks. IEEE Trans. Parallel Distrib. Syst..

[8] Yuen K., Liang B., Baochun L. (2008). A Distributed Framework for Correlated Data Gathering in Sensor Networks. IEEE Trans. Veh. Technol..

[9] Ciancio A.G., Pattem S., Ortega A., Krishnamachari B. Energy-Efficient Data Representation and Routing for Wireless Sensor Networks Based on a Distributed Wavelet Compression Algorithm. Proceedings of the 5th International Conference on Information Processing in Sensor Networks.

[10] Zheng H., Yang F., Tian X., Gan X., Wang X., Xiao S. (2015). Data Gathering with Compressive Sensing in Wireless Sensor Networks: A Random Walk Based Approach. IEEE Trans. Parallel Distrib. Syst..

[11] Hwang S., Ran R., Yang J., Kim D.K. (2016). Multivariated Bayesian Compressive Sensing in Wireless Sensor Networks. IEEE Sens. J..

[12] Eldar Y., Kutyniok G. (2012). Compressed Sensing: Theory and Applications.

[13] Luo C., Wu F., Sun J., Chen C.W. Compressive data gathering for large-scale wireless sensor networks. Proceedings of the International Conference on Mobile Computing and NETWORKING.

[14] Luo C., Wu F., Sun J., Chen C.W. (2010). Efficient Measurement Generation and Pervasive Sparsity for Compressive Data Gathering. IEEE Trans. Wirel. Commun..

[15] Caione C., Brunelli D., Benini L. (2012). Distributed Compressive Sampling for Lifetime Optimization in Dense Wireless Sensor Networks. IEEE Trans. Ind. Inform..

[16] Xiang L., Luo J., Vasilakos A. Compressed Data Aggregation for Energy Efficient Wireless Sensor Networks. Proceedings of the 8th Annual IEEE Communications Society Conference on Sensor, Mesh and Ad Hoc Communications and Networks.

[17] Xie R., Jia X. (2014). Transmission-Efficient Clustering Method for Wireless Sensor Networks Using Compressive Sensing. IEEE Trans. Parallel Distrib. Syst..

[18] Xue T., Dong X., Shi Y. (2013). Multiple Access and Data Reconstruction in Wireless Sensor Networks Based on Compressed Sensing. IEEE Trans. Wirel. Commun..

[19] Chen W., Wassell I.J. (2016). Optimized Node Selection for Compressive Sleeping Wireless Sensor Networks. IEEE Trans. Veh. Technol..

[20] Masoum A., Meratnia N., Havinga P.J.M. (2013). A Distributed Compressive Sensing Technique for Data Gathering in Wireless Sensor Networks. Proc. Comput. Sci..

[21] Lindberg C., Amat A.G.I., Wymeersch H. (2017). Compressed Sensing in Wireless Sensor Networks without Explicit Position Information. IEEE Trans. Signal Inform. Proc. Over Netw..

[22] Li S., Xu L.D., Wang X. (2013). Compressed Sensing Signal and Data Acquisition in Wireless Sensor Networks and Internet of Things. IEEE Trans. Industr. Inform..

[23] Yin M., Yu K., Wang Z. (2016). Compressive Sensing Based Sampling and Reconstruction for Wireless Sensor Array Network. Math. Prob. Eng..

[24] Donoho D.L., Tsaig Y., Drori I., Starck J. (2012). Sparse Solution of Underdetermined Systems of Linear Equations by Stagewise Orthogonal Matching Pursuit. IEEE Trans. Inf. Theory.

[25] Needell D., Tropp J.A. (2008). CoSaMP: Iterative Signal Recovery From Incomplete and Inaccurate Samples. Appl. Comput. Harmonic Anal..

[26] Do T.T., Gan L., Nguyen N.P., Tran T.D. In Proceedings of the Sparsity Adaptive Matching Pursuit Algorithm for Practical Compressed Sensing.

[27] Wang J., Tang S., Yin B., Li X.Y. (2012). Data gathering in wireless sensor networks through intelligent compressive sensing. Proc. IEEE Infocom..

[28] Fragkiadakis A., Charalampidis P., Tragos E. Adaptive compressive sensing for energy efficient smart objects in IoT applications. Proceedings of the International Conference on Wireless Communications, Vehicular Technology, Information Theory and Aerospace & Electronic Systems.

[29] Candes E.J., Romberg J.K., Tao T. (2006). Robust uncertainty principles: Exact signal reconstruction from highly incomplete frequency information. IEEE Trans. Inf. Theory.

[30] Candes E.J., Tao T. (2005). Decoding by linear programming. IEEE Trans. Inf. Theory.

[31] Donoho D.L. (2006). Compressed sensing. IEEE Trans. Inf. Theory.

[32] Aharon M., Elad M., Bruckstein A. (2006). K-SVD: An Algorithm for Designing Overcomplete Dictionaries for Sparse Representation. IEEE Trans. Signal Process..

[33] Candes E.J., Tao T. (2006). Near-Optimal Signal Recovery From Random Projections: Universal Encoding Strategies?. IEEE Trans. Inf. Theory.

[34] Haupt J., Bajwa W.U., Rabbat M., Nowak R. (2008). Compressed Sensing for Networked Data. IEEE Signal Process. Mag..

[35] Malioutov D.M., Sanghavi S., Willsky A.S. Compressed Sensing With Sequential Observations.

[36] Zhang M., Wen Y., Chen J., Yang X., Gao R., Zhao H. (2018). Pedestrian Dead-Reckoning Indoor Localization Based on OS-ELM. IEEE Access.

[37] Davenport M.A., Wakin M.B. (2010). Analysis of Orthogonal Matching Pursuit Using the Restricted Isometry Property. IEEE Trans. Inf. Theory.

